# The Role of Stimulus Salience and Attentional Capture Across the Neural Hierarchy in a Stop-Signal Task

**DOI:** 10.1371/journal.pone.0026386

**Published:** 2011-10-17

**Authors:** Carsten N. Boehler, Lawrence G. Appelbaum, Ruth M. Krebs, Ling-Chia Chen, Marty G. Woldorff

**Affiliations:** 1 Center for Cognitive Neuroscience, Duke University, Durham, North Carolina, United States of America; 2 Department of Experimental Psychology, Ghent University, Ghent, Belgium; 3 Department of Psychiatry, Duke University, Durham, North Carolina, United States of America; Katholieke Universiteit Leuven, Belgium

## Abstract

Inhibitory motor control is a core function of cognitive control. Evidence from diverse experimental approaches has linked this function to a mostly right-lateralized network of cortical and subcortical areas, wherein a signal from the frontal cortex to the basal ganglia is believed to trigger motor-response cancellation. Recently, however, it has been recognized that in the context of typical motor-control paradigms those processes related to actual response inhibition and those related to the attentional processing of the relevant stimuli are highly interrelated and thus difficult to distinguish. Here, we used fMRI and a modified Stop-signal task to specifically examine the role of perceptual and attentional processes triggered by the different stimuli in such tasks, thus seeking to further distinguish other cognitive processes that may precede or otherwise accompany the implementation of response inhibition. In order to establish which brain areas respond to sensory stimulation differences by rare Stop-stimuli, as well as to the associated attentional capture that these may trigger irrespective of their task-relevance, we compared brain activity evoked by Stop-trials to that evoked by Go-trials in task blocks where Stop-stimuli were to be ignored. In addition, region-of-interest analyses comparing the responses to these task-irrelevant Stop-trials, with those to typical relevant Stop-trials, identified separable activity profiles as a function of the task-relevance of the Stop-signal. While occipital areas were mostly blind to the task-relevance of Stop-stimuli, activity in temporo-parietal areas dissociated between task-irrelevant and task-relevant ones. Activity profiles in frontal areas, in turn, were activated mainly by task-relevant Stop-trials, presumably reflecting a combination of triggered top-down attentional influences and inhibitory motor-control processes.

## Introduction

Inhibitory motor control — i.e. the ability to suppress unwanted behavioral responses — provides crucial flexibility in goal-directed behavior, allowing individuals to quickly adjust to a changing environment and to overcome pre-potent responses when they are inadequate or inappropriate (see [1] for a review). Interest in this topic has dramatically increased over the past several years in accord with the central role of this function in normal human behavior and development, as well as in a range of neurological and psychiatric conditions, such as attention-deficit hyperactivity disorder and substance abuse [2]–[6].

One of the most prominent experimental paradigms designed to investigate response-inhibition capabilities is the Stop-signal task [7], [8]. In this task, a choice-reaction Go-stimulus is rapidly followed, on a minority of trials, by a Stop-stimulus requiring participants to withhold the response to the Go-stimulus. Variants of this and related tasks have been used extensively with a variety of methodological approaches to investigate brain processes underlying response inhibition. Converging evidence from these studies has led to the view that a mostly right-hemisphere network of brain areas plays a critical role in response inhibition (but see [9]). This network includes the inferior frontal gyrus (IFG; especially the frontal operculum extending into the insula) and the pre-supplementary motor area (pre-SMA), which in turn interacts with the basal ganglia and the thalamus (for reviews see [4], [5], [10]).

Although it is very likely that the loop between the frontal cortex and the basal ganglia/thalamus described above is a core structure subserving response inhibition, it is increasingly recognized that other mechanisms play an important role leading up to response inhibition and in determining whether it will be successful or not. Specifically, it has been reported that selective attention to the task-relevant stimuli can play an important role in determining trial outcome in the Stop-signal task. Numerous studies have reported transient modulations of sensory processing of the relevant stimuli in the time-range of the sensory evoked N1 ERP component, which precedes the implementation of response inhibition, and that these modulations are predictive of the outcome of the process [11]–[15]. Due to the timing and the posterior topography, such effects can be compellingly attributed to differences in sensory processing, including due to attentional modulations of that processing.

Unfortunately, such conclusions are much more difficult to draw for activity at later time-ranges and in other brain areas, so that a separation of perceptual/attentional processes from those that are directly related to response inhibition has proven difficult. A case in point relates to the right IFG, which has received a lot of experimental support as a key structure in response inhibition. This area is reliably activated in human fMRI studies investigating response inhibition (for a recent comprehensive review, see [10]), while lesion and electrophysiological studies in humans have provided corroborating evidence [16]–[20]. Recent studies, however, have challenged the view that the right IFG is directly involved in response inhibition (but see [21]). For example, strong right IFG activations have also been reported in response to other rare stimuli besides task-relevant Stop-stimuli ([22]–[24], see also [25]–[29]), consistent with its reported participation in the ventral attention system that has been implicated in bottom-up attentional processes triggered relatively automatically by salient environmental events [30], [31].

An important distinction in this context that has not yet been established (neither for the right IFG nor for other involved brain areas) is the degree of automaticity with which Stop-trial stimulation elicits neural activity. Specifically, in the existing attention literature it is appreciated that the presentation of rare, and/or physically salient, stimuli (note that Stop-trials meet both criteria) tend to automatically capture attention and activate at least parts of the ventral attention system [30], [32]. Such processes can even occur if these stimuli are entirely task-irrelevant (for a recent discussion, see [33]). In the context of the Stop-signal task, however, the degree to which neural activity is related to such salience-triggered processes is not clear, versus how much such activation may depend on the general task context, in which the behavioral relevance of stimuli other than Go-stimuli needs to be determined. Establishing such a distinction is important in order to gain insights into which areas and processes are under active top-down control during a Stop-signal task, even if their function is related to control processes that are not directly related to response inhibition. Moreover, because these other functions could also be derailed in psychopathology, thereby potentially mimicking deficits directly in motor control (e.g., [34]), disentangling and understanding these processes better is an important goal.

**Figure 1:**
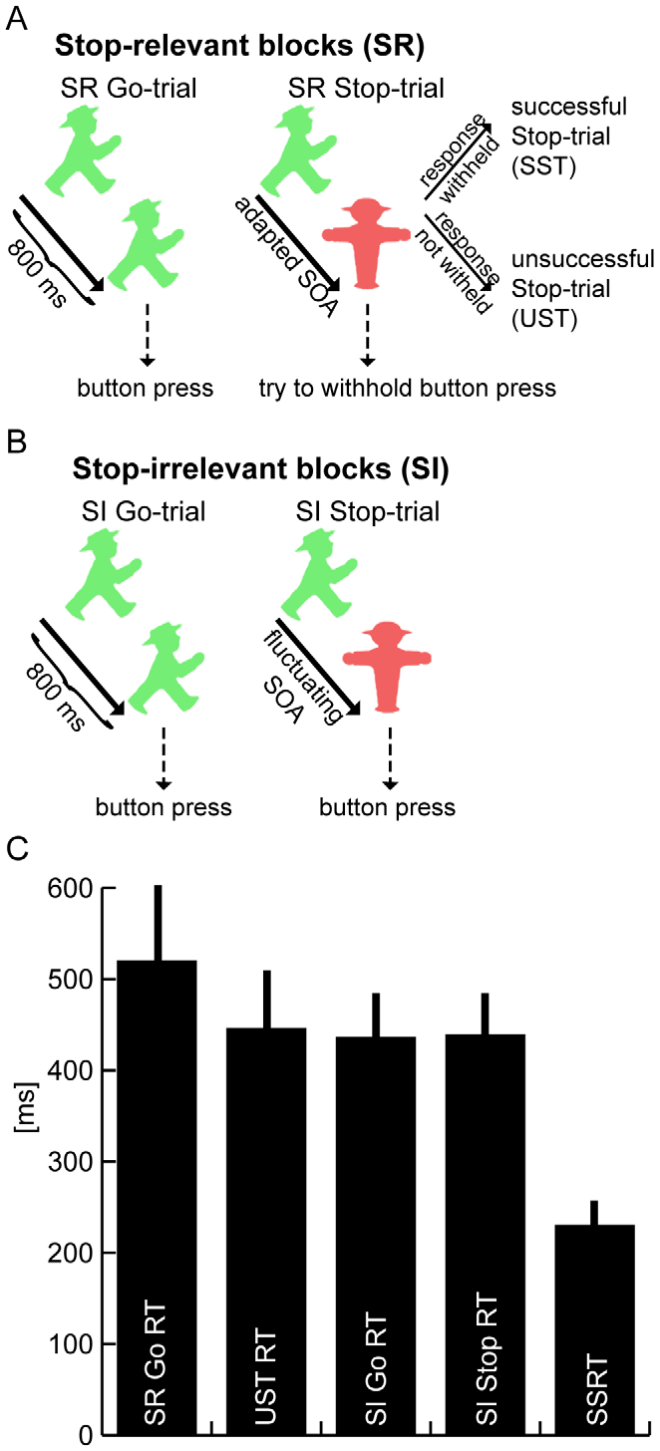
Paradigm and behavioral data. (A) In Stop-relevant blocks, a choice-reaction stimulus (a green German traffic-light symbol oriented to the left or right) was either presented for the entire stimulus duration of 800 ms (Go-trial) or replaced by a red Stop-stimulus (Stop-trial) after a variable SOA set trial-to-trial by a tracking algorithm. The Stop-stimulus indicated that the response to the Go-stimulus was to be cancelled, yielding successful (SSTs) and unsuccessful Stop-trials (USTs). (B) In Stop-irrelevant blocks the visual stimulation was identical, but the Stop-stimuli were all irrelevant, i.e. responses were required for all the Go-trials regardless of whether they were followed by a Stop-stimulus. (C) Response times were slowest for Stop-relevant (SR) Go-trials but similar for unsuccessful Stop-trial, Stop-irrelevant (SI) Stop-trials, and Stop-irrelevant Go-trials. The Stop-signal reaction time (SSRT) was calculated to be 230 ms (grand-average data + standard error of the mean (SEM)).

In the present report, we have carried out additional sets of analyses of the data from a recent study [35] that included, as a control condition, task-irrelevant Stop-trials from separate task blocks (see Fig. 1). In our previous report, this control condition was used specifically to subtract out activity related to the sensory processing of Stop-stimuli. Here, we expanded our analyses of these data in order to gauge, on a brain-wide level, the degree to which activity in different brain areas is related to the sensory and attention-attracting features of Stop-stimuli. Additionally, we performed an ROI analyses to investigate the relative degree of activation by Go-trials, task-irrelevant Stop-trials, and task-relevant Stop-trials in key brain areas. These analyses provide activity profiles indicative of separable neural operations that have important implications towards a better understanding of the specific systems-level neural circuits that lead to and implement response inhibition.

## Methods

### Participants and Ethics Statement

Eighteen participants took part in this study, two of which had to be excluded due to technical problems, and another one due to particularly poor behavioral performance. The 15 remaining participants (nine female) had a mean age of 22.9 years, all with correct or corrected-to-normal visual acuity, and none reporting a history of psychiatric or neurological disorders. All participants gave written informed consent and the study was approved by the Duke University Health System Institutional Review Board. Participants were compensated $20 per hour.

### Task

The present experiment entailed two variants of the typical Stop-signal task [36] that differed only in the instructions given to the participants. During Stop-relevant blocks, participants were instructed to try to withhold their response when a Stop-stimulus followed a Go-stimulus, whereas in Stop-irrelevant blocks the visual stimulation was identical, but participants were instructed to ignore the Stop-stimuli and thus to respond to all Go-stimuli irrespective of whether they were followed by a Stop-stimulus [18]. Each task was performed once per experimental run (approximately 2.5 minutes each), separated by a 16-sec break (i.e., task break, with continuing MR data acquisition). Odd runs began with the Stop-relevant task, followed by the Stop-irrelevant task, with even runs in the opposite order. Ten runs were collected for each participant, yielding a total of 943 trials across all conditions per participant. The time between trial onsets was varied pseudo-randomly between 2 and 8 seconds (gamma distribution; average 3.2 sec) to allow for the separation of different conditions in an event-related fMRI analysis [37].

### Stop-relevant blocks

Stop-relevant blocks used a standard Stop-signal task (using German traffic-light signs, see Fig. 1A), entailing a random sequence of frequent Go-trials and less-frequent Stop-trials. On Go-trials (80% of all trials), only a Go-stimulus was presented, requiring a rapid choice response. On Stop-trials (20% of trials), the Go-stimulus was followed shortly after by the presentation of a Stop-stimulus, indicating that the response to the Go-stimulus was to be canceled. On Go-trials, a green symbol was presented for 800 ms, and participants had to decide whether it was oriented to the left or right (mapped to the right index and middle finger). Stop-trials started identically, but after a variable stimulus onset asynchrony (SOA) the Go-stimulus was replaced by a red Stop-stimulus until the end of the total stimulus duration of 800 ms. The SOA between the Go- and the Stop-stimulus is an important determinant for whether participants are able to withhold the response to the Go-stimulus (successful Stop-trials, **SST**) or not (unsuccessful Stop-trials, **UST**; see [8]). Note that for discussion of the SST and UST trials below, the respective block is usually not specified because these conditions are exclusive to the Stop-relevant blocks.

A common approach for controlling performance is to titrate the Go-Stop SOA using an adaptive staircase procedure to yield approximately equivalent numbers of SST and UST for each participant. We implemented such a procedure here, increasing the SOA by 17 ms (one refresh screen) after SSTs and decreasing it by the same amount after USTs (starting SOA: 200 ms). This procedure allowed us to calculate the Stop-signal response time (SSRT), which is viewed as reflecting the mean amount of time that is required to implement the inhibition of a motor response and is derived by subtracting the mean Go-Stop SOA from the average Go-trial response time [8].

### Stop-irrelevant blocks

During Stop-irrelevant blocks, visual stimulation was identical to the Stop-relevant ones (Fig. 1B), but participants were instructed to respond to all Go-stimuli irrespective of the occurrence of Stop-stimuli. To equate the sensory stimulation as much as possible between the two block types, we also varied the Go-Stop SOA during Stop-irrelevant blocks. Specifically, the SOA value resulting from the staircase procedure of the preceding Stop-relevant block was used as the initial value, which was then varied in a random one-up/one-down fashion after each Stop-trial, staying within +/- three 17-ms steps of the initial value. Stop-relevant blocks used the end value of the preceding Stop-relevant-block staircase as their starting value.

### Data acquisition and basic analysis

MR data was acquired on a 3-Tesla GE Signa MRI system. Functional images were acquired with a reverse spiral imaging sequence (TR = 2000 ms, TE = 25 ms; flip angle  = 75°; 32 slices with 3×3×3 mm resolution; AC-PC orientation providing coverage approximately from the top of the brain down to the pons). The first five functional images were excluded from the analysis, to allow the scanner to reach steady-state magnetization. For anatomical reference, a high-resolution structural T1 (3D Fast Spoiled Gradient Recalled (FSPGR); 1×1×1 mm resolution) was obtained. The fMRI data were analyzed using SPM5 (http://www.fil.ion.ucl.ac.uk/spm/). All functional images were corrected for acquisition time delay, spatially realigned, and normalized by applying the normalization parameters used to warp the high-resolution T1 image to the SPM template. Images were resliced to a voxel size of 2×2×2 mm and smoothed with an isotropic 8-mm full-width half-maximum Gaussian kernel. For each participant, a statistical model was computed by applying a canonical hemodynamic response function (HRF) combined with time and dispersion derivatives for each of the conditions, including a 128-sec high-pass filter [38]. All conditions were modeled separately, restricting the analyses on the trials with correct responses (or with a successfully withheld response in the case of successful Stop-trials). Additional regressors were included to model trials with incorrect responses, misses, and break onsets, as well as for modeling the six realignment parameters measuring the participants’ movements during the experiment. For visualization purposes, activation maps were rendered on the SPM single-subject template.

### Data analysis

The parameter estimates resulting from each condition/contrast and participant (first-level analysis) were entered into a second-level, random-effects group analysis using one-sample t-tests. In order to test for areas that were more active for Stop-irrelevant Stop-trials than Stop-irrelevant Go-trials on a brain-wide level, a voxel-wise analysis was performed. The respective group-level results were thresholded at T>3 (uncorrected) and a minimum cluster size of k = 10 contiguous voxels. Additionally, cluster-level correction for multiple comparisons was performed. Clusters surviving this correction (p<0.05) are highlighted in the Results tables, and strong inferences are limited to these areas. Despite the danger of false positives, we also report those activations that did not survive this correction. Such two-stage procedure was employed to meet our inferential goals to simultaneously not *underestimate* activity differences in areas that are typically associated with the Stop-signal task (i.e., to not make strong claims about the absence of activity differences based on a highly conservative threshold), while also highlighting which activations are quite certainly not false positives.

Additionally, a region of interest (ROI) analysis was performed to compare activity elicited by the different conditions in the key regions involved in this task. In order to define ROIs that would allow for a comparison between Stop-trials from the Stop-relevant and the Stop-irrelevant task blocks, a t-contrast was employed that tested the average of these Stop-trial responses across the blocks against the average of all Go-trial responses across the blocks. Due to the very robust and widespread activations identified by this contrast, the group-level effects were thresholded comparatively conservatively (p<0.01; FDR-corrected corrected on the voxel level with an extent threshold k = 50 contiguous voxels; note that the resulting clusters also survived cluster-level multiple-comparison correction). Defining ROIs on the basis of this contrast enabled quantitative comparisons between the Stop-relevant and Stop-irrelevant Stop-trials in these regions, because the ROI selection was not biased in favor of either of those conditions [39]. Ten 4-mm-radius spherical ROIs were selected (see Results). Marsbar (http://marsbar.sourceforge.net/) was used to extract percent-signal-change values from these ROIs. Statistical assessment of the ROI data and the behavioral data was accomplished using repeated-measures analyses of variance (rANOVAs), with non-sphericity correction of the degrees of freedom (Greenhouse-Geisser algorithm) where necessary, or using paired t-tests reporting two-tailed p-values if not indicated otherwise. For the ROI analysis, our inferential goals also had an influence on the choice of statistical significance criterion. Specifically, p-values are reported without correction for multiple comparisons. Although this risks some false-positive results, applying strict correction would have artificially biased our results towards the conclusion that there are no differences between Stop-trials from the two task-blocks (as well as between SST and UST). Nevertheless, we note that some differences could represent false positives and need to be interpreted with a degree of caution.

## Results

### Behavioral Results

Participants performed very accurately during both the Stop-relevant and Stop-irrelevant task blocks. No significant differences in accuracy were observed for the three trial types that always required a response (i.e., Stop-relevant Go-trials [97.6%], Stop-irrelevant Go-trials [96.6%], and Stop-irrelevant Stop-trials [97.1%]; F(1.5,21.4) = 2.2, p = 0.15). Response times were slower on Stop-relevant Go-trials (520 ms) relative to unsuccessful Stop-trials (446 ms) and relative to Stop-irrelevant Go- and Stop-trials (436 and 439 ms; overall F-Test: F(1.8,25.3) = 32.3, p<0.001), but were similar between the latter three conditions (F(1.3,17.61) = 0.9, p = 0.37; see Fig. 1C). During Stop-relevant Stop-trials, participants managed to withhold their behavioral response on approximately half of the trials (52.7%), indicating the success of our staircase SOA-adjustment procedure. The average SSRT across subjects was 230 ms.

### fMRI Results

#### Activity related to Stop-irrelevant Stop-trials

**Figure 2:**
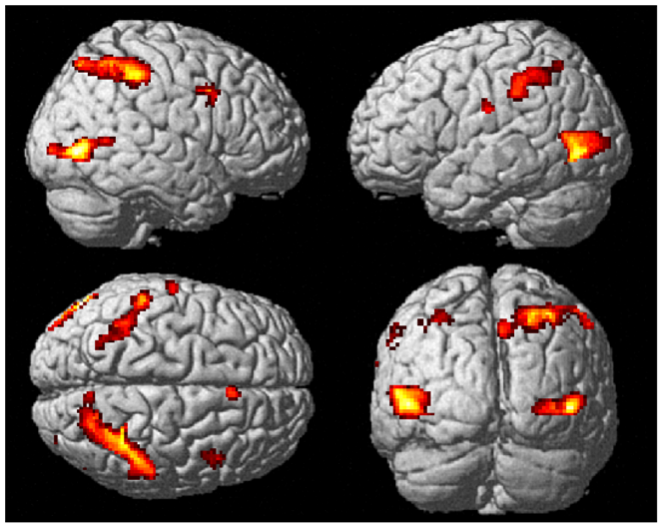
Grand-average comparison of Stop-trials versus Go-trials from the Stop-irrelevant task blocks (activation maps thresholded at T>3 (uncorrected) and cluster size k>10). Activity differences were most prominent in occipito-temporal and parietal areas but were also present in the right IFJ and pre-SMA (note that only the large parietal and occipital clusters survived strict cluster-level correction for multiple comparisons).

In order to identify brain areas that respond differentially to Stop-stimuli, as compared to Go-stimuli, even if those stimuli are entirely task-irrelevant, we performed a voxel-wise comparison between Stop-trials and the Go-trials in the Stop-irrelevant blocks (T>3; k = 10; additionally, cluster-level correction for multiple comparison was employed, and clusters surviving this procedure are highlighted below and in table 1). Differences were not only found in lateral occipital areas, as can be expected based on the differences in sensory stimulation between these trial types, but also bilaterally in widespread clusters in the inferior parietal lobules (IPL; Fig. 2 and Table 1). Importantly, these main posterior clusters all survived multiple-comparison correction on the cluster level. Turning to the frontal cortex, two clusters were identified, one in the right IFJ, and another one in the right pre-SMA. Importantly, these clusters were too weak/small to survive the multiple-comparison correction employed. Although these could reflect false-positive results, the locations of these clusters are well in line with typical activations in the Stop-signal task in these areas (see also ROI analysis below), thus giving some more credibility to both effects. Due to the failure to reach cluster-level-corrected significance, however, these activations need to be interpreted cautiously. Taken together, activity that is purely triggered by the perceptual and attention-attracting aspects of Stop-trials are not limited to ventral sensory areas, but are also present in inferior parietal areas, with possible contributions from the right IFJ and pre-SMA.

**Table 1:** fMRI activations for the contrast “Stop-irrelevant Stop-trials vs. Stop-irrelevant Go-trials”.

Anatomical structure	Hemi-sphere	Cluster size [voxel]	T-Value	Peak coordinates MNI (mm)x y z
*Frontal cortex*				
Inferior frontal junction (IFJ)	R	124	4.73	42 8 36
Pre-SMA	R	83	4.22	4 20 48
				
*Parietal cortex*				
Inferior parietal lobule (IPL)*	R	1651	6.37	30 -56 50
Inferior parietal lobule (IPL)*	L	647	5.06	-54 -34 36
Supramarginal gyrus	L	52	4.18	-62 -18 26
				
*Occipital cortex*				
Middle occipital gyrus (MOG)*	L	576	8.51	-50 -76 0
Middle occipital gyrus (MOG)*	R	750	7.15	46 -72 0

Main local maxima. Data are thresholded at T>3 (uncorrected), with a cluster-level of k = 10.

(*) denotes clusters that are significant after correction for multiple comparisons on the cluster level.

#### ROI selection and predicted activity profiles

While the above analyses provide a formal brain-wide test for which areas are activated during Stop-trials (as compared to Go-trials) even when these stimuli are task-irrelevant, the relationship to activity triggered by task-relevant Stop-trials is hard to evaluate without direct reference to these other trial types. Importantly, activity in some areas might not be triggered in an all-or-none fashion by Stop-stimuli. Rather it is possible that some areas may be activated in a graded fashion, wherein a certain amount of activity is triggered even by task-irrelevant Stop-stimuli, which gets more pronounced if those stimuli are in fact task-relevant. A good example process for which such a pattern might be present is attentional capture, although other processes might also be engaged in a graded fashion. Specifically, attentional capture has an automatic component that does not depend on task-relevance. However, attentional capture effects can get enhanced if the capturing stimulus is furthermore relevant to the task. Accordingly, in order to provide a more detailed analysis of the contributions of different key areas to the processing of task-relevant and task-irrelevant Stop-trials, we performed additional analyses within ROIs that were delineated based on both kinds of Stop-trials. Another advantage of such an ROI analysis is that voxel-wise comparisons are necessarily quite conservative, whereas ROI-analyses can focus on the most relevant areas derived from orthogonal contrasts, thus ameliorating the multiple-testing problem. In order to allow for an unbiased comparison between the Stop-trials from the different task blocks, ROIs were selected on the basis of a contrast comparing all Stop-trials from the two task blocks (i.e., Stop-relevant and Stop-irrelevant) against all Go-trials from those tasks. The present ROI analysis is related to an ROI analysis of some of these data applied in our earlier paper [35]. As compared to this earlier report, however, we used the Stop-irrelevant Stop-trials here as an active part of the ROI-defining contrast instead of using it as a baseline condition. Moreover, our earlier report used a conjunction of SST and UST for the ROI definition (rather than their average), which could have introduced some bias towards finding similar activity estimates for the two conditions in the subsequent analysis. Such bias would be avoided with the present analysis.

**Figure 3:**
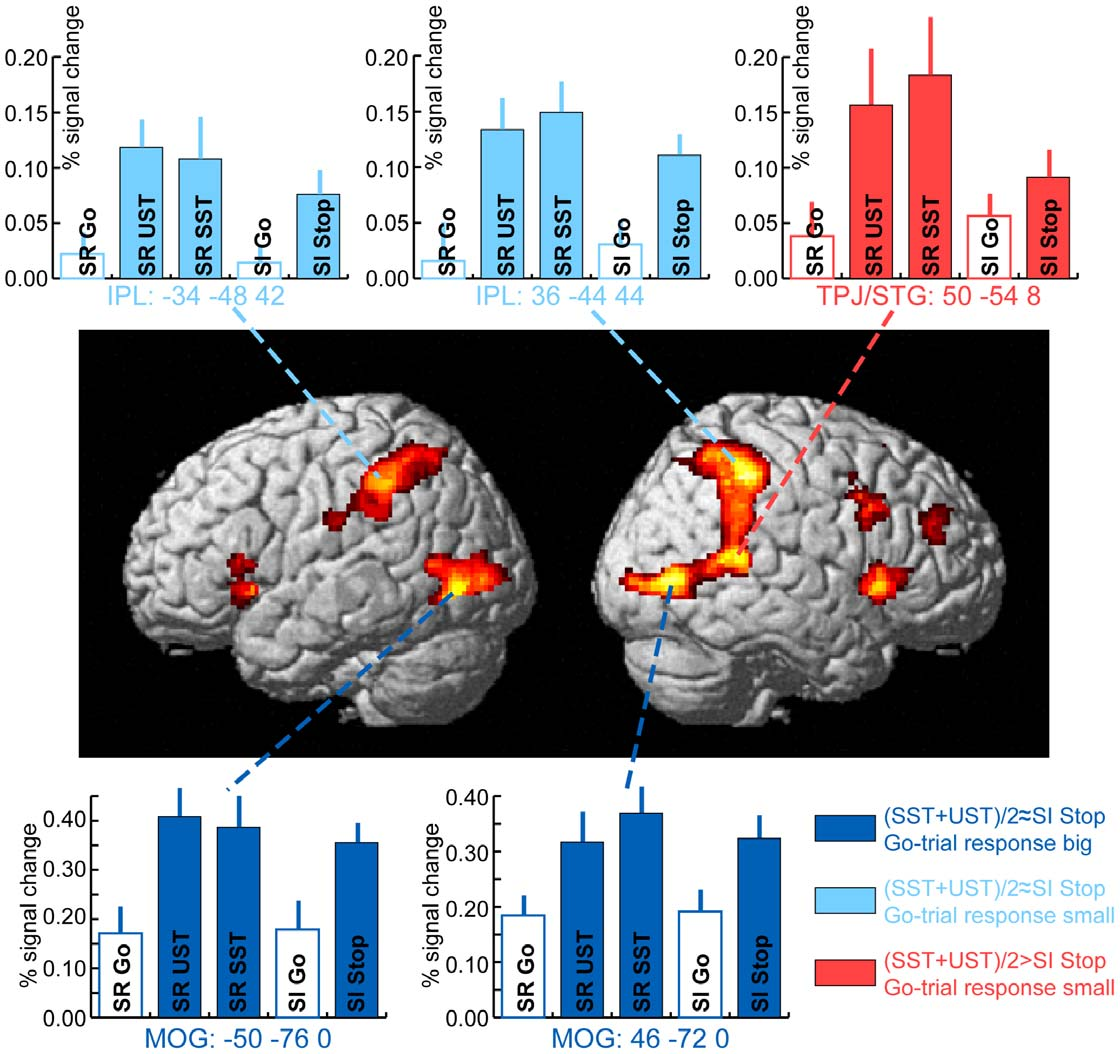
Grand-average activity estimates in posterior brain areas for the comparison of all Stop-trials in the two tasks (average of Stop-relevant (SR) and Stop-irrelevant (SI)) versus the average of all the Go-trials in the two tasks (MNI coordinates; activation maps thresholded at p<0.01 (FDR-corrected) and cluster size k>50). Areas in the lateral occipital cortex displayed a pattern of activity that mostly reflected sensory stimulation (i.e., no significant difference between task-relevant [average of SST and UST] and task-irrelevant Stop-trials, along with substantial response to Go-trials; dark blue bars). Bilateral responses in the inferior parietal lobule appeared to mainly reflect attentional capture by the infrequent Stop-stimulus, irrespective of its task relevance (i.e., no significant difference between task-relevant [average of SST and UST] and task-irrelevant Stop-trials, accompanied by a weak response to Go-trials; light blue bars). The only area in the posterior part of the brain that reflected the task-relevance of Stop-stimuli was in the superior temporal gyrus (STG) close to the TPJ (red bars; significantly larger response to Stop-relevant Stop-trials [average of SST and UST] than Stop-irrelevant Stop-trials, along with a weak response to Go-trials). Error bars depict the SEM; activity estimates for Go-trials are represented without a fill color to set them apart from Stop-trials and to indicate that the ROI definition favored Stop-trials so that statistical comparisons including Go-trials were avoided.

**Figure 4:**
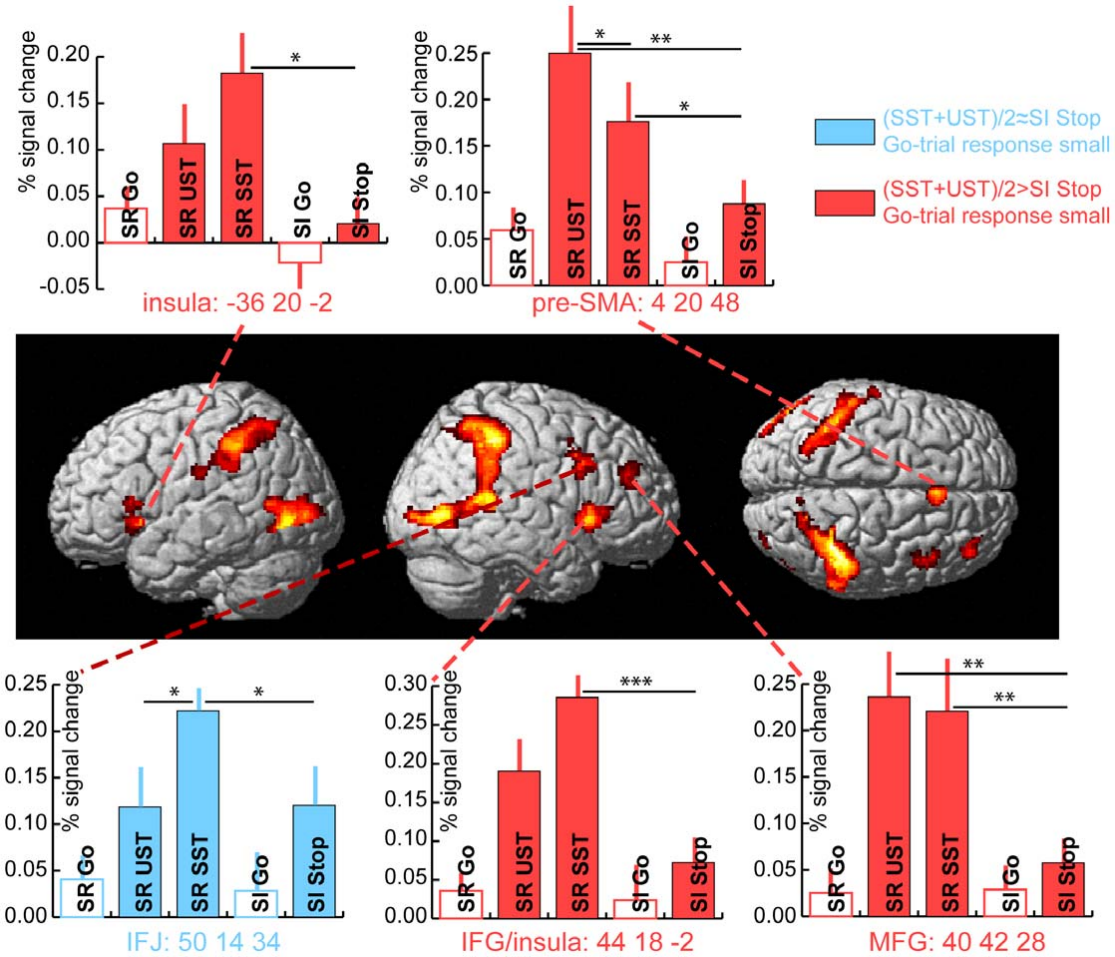
Grand-average activity estimates in frontal brain areas for the comparison of all Stop-trials (average of Stop-relevant (SR) and Stop-irrelevant (SI)) versus the respective Go-trials (MNI coordinates; activation maps thresholded at p<0.01 (FDR-corrected) and cluster size k>50). None of the frontal areas displayed strong activity estimates for Go-trials. All frontal areas except for IFJ displayed a clear difference between the average response to the task-relevant Stop-trials and the response to the task-irrelevant ones (red bars). Additional significant differences between the individual Stop-trial types are indicated in the bar plots (*<0.05; **<0.01; ***<0.001; two-tailed; error bars depict the SEM.). Right IFJ displayed a somewhat different pattern, in that SST responses were larger than both the UST and Stop-irrelevant Stop-trial responses, but that the average response to Stop-relevant Stop-trials (i.e., averaged across SSTs and USTs) was not larger than that to Stop-irrelevant ones. Error bars depict the SEM; activity estimates for Go-trials are represented without a fill color to set them apart from Stop-trials and to indicate that the ROI definition favored Stop-trials so that statistical comparisons including Go-trials were avoided.

Due to the very robust and widespread activations that were identified by this contrast, we opted for a comparatively conservative voxel-level threshold (FDR corrected p<0.01; k = 50). This contrast yielded eight activation clusters (note that all clusters furthermore survived cluster-level correction for multiple comparisons; note also that the present set of areas is very similar to other studies that have compared Stop-trials with Go-trials, which presumably indicates that activity levels in Stop-relevant Stop-trials were sufficient to identify typical stopping-related areas even when averaged with Stop-irrelevant Stop-trials that may have failed to elicit substantial activity in some of these areas.). The respective maxima of seven of these were highly distinctive and were thus directly used for further analysis (see Figs. 3, 4 and Table 2). Five of these were found in frontal cortex, including right-hemispheric lateral frontal areas IFG (protruding into the anterior insula) and the inferior frontal junction (IFJ). Additionally, this contrast revealed activity in a middle frontal gyrus (MFG) area, along with right pre-SMA and the left anterior insula. In the left hemisphere two additional substantial clusters were found, namely in the lateral middle occipital gyrus (MOG) and in the inferior parietal lobule (IPL). A final eighth cluster was identified in the posterior part of the right hemisphere, but seemed to be a grouping of three subclusters (see Figs. 3, 4). Accordingly, the three main local maxima in this cluster were each analyzed separately (right MOG, right IPL, and a cluster in the superior temporal gyrus close to the right temporo-parietal junction (TPJ/STG), yielding 10 locations total. (Note that we will use the combined abbreviation TPJ/STG here because the present local maximum is a bit ventral to the typical TPJ location. However, a slightly more dorsal local maximum displayed a very similar activity pattern. Moreover, there is some heterogeneity between studies reporting activity in TPJ (see e.g., [30]), so that the present activation seems quite likely to relate to the functions that tend to be ascribed to the right TPJ.) Percent signal change values were determined for spherical ROIs around these ten maxima (see Methods; also see [35], for other functional contrasts of data from this experiment).

**Table 2:** fMRI activations for the contrast “all Stop-trials vs. all Go-trials” (average Stop-relevant and Stop-irrelevant blocks).

Anatomical structure	Hemi-sphere	Cluster size [voxel]	T-Value	Peak coordinates MNI (mm)x y z
*Frontal cortex*				
Inferior frontal gyrus (IFG)/anterior insula	R	393	8.16	44 18 -2
Inferior frontal junction (IFJ)	R	259	7.31	50 14 34
Middle frontal gyrus (MFG)	R	95	7.24	40 42 28
Pre-SMA	R	300	6.46	4 20 48
Anterior insula	L	274	6.15	-36 20 -2
*Parietal cortex*				
Inferior parietal lobule (IPL)	R	3613#	8.62	36 -44 44
Inferior parietal lobule (IPL)	L	1471	7.06	-34 -48 42
*Temporal cortex*				
Temporo-parietal junction/ superior temporal gyrus (TPJ/STG)	R	3613#	8.21	50 -54 8
*Occipital cortex*				
Middle occipital gyrus (MOG)	L	754	10.01	-50 -76 0
Middle occipital gyrus (MOG)	R	3613#	9.06	46 -72 0

Main local maxima. Data are thresholded at p<0.01 (FDR-corrected), with a cluster-level of k = 50.

(^#^) the three main local maxima were taken from this larger cluster subtending the right occipito-temporal and parietal cortex.

Among these ROIs, we predicted finding three distinctive activity profiles for the different conditions: (1) Sensory-driven activity that would be present for Go-trials and further enhanced for Stop-trials (due to the extra sensory stimulation), but not differing significantly between Stop-relevant and Stop-irrelevant Stop-trials (dark blue bars in Fig. 3). (Note that the ROI selection favored Stop-trials, as it is based on a direct comparison of Stop-trials with Go-trials. Therefore, statistical tests between Stop- and Go-trials within the ROIs were avoided in our analyses here. Similarly, tests comparing activity estimates for Go-trials against zero were also not performed, and the respective results are only displayed to serve as an approximate reference and for qualitative comparisons between the activity profiles in different areas. To highlight this fact and to further set them apart from Stop-trials, bargraphs referring to Go-trials are represented without a fill color in Fig. 3 and 4.) (2) Activity associable with automatic attentional capture by the rare Stop-stimuli, showing little or no activity on Go-trials but strong responses on Stop-trials irrespective of their task relevance (i.e., not differing significantly for stop-irrelevant versus stop-relevant Stop-trials; light blue bars in Fig. 3, 4). (3) Activity profiles dominated by responses to the Stop-relevant Stop-stimuli, with significantly stronger responses to task-relevant than task-irrelevant Stop-trials and little or no response to Go-trials (red bars in Fig. 3, 4). This activity pattern would be indicative of top-down control processes in response to the Stop-stimuli, including both directed attention toward them due to their relevance and response inhibition following their detection.

#### Posterior brain regions

Analyses of the five posterior ROIs revealed three different activity profiles that were largely symmetrical for the bilaterally activated areas (Fig. 3). Occipital ROIs (left and right MOG) revealed a pattern in line with simple visual processing, in that activity estimates were similarly prominent for both types of Go-trials (i.e., in both the Stop-relevant and Stop-irrelevant task blocks), but were substantially larger for Stop-trials of either task block, reflecting the presentation of an additional salient stimulus on all Stop-trials. In contrast, the bilateral IPL regions produced very little activity for either kind of Go-trial, but showed strong responses for all Stop-trials. These IPL activations, however, did not differ significantly between the Stop-relevant and Stop-irrelevant Stop-trials (p>0.1 in both hemispheres). Finally, the TPJ/STG ROI also displayed little activity related to Go-trials from either trial block type. More importantly, however, this area yielded a statistically significant difference between the average of Stop-relevant Stop-trials vs. Stop-irrelevant Stop-trials (t(14) = 2.2; p = 0.04), distinguishing this area from all other posterior regions.

#### Frontal brain regions

Of the five frontal clusters identified, all areas except the right IFJ displayed qualitatively the same pattern of activity. More specifically, these areas did not respond strongly to Go-trials in either task block, nor to the Stop-trials from the Stop-irrelevant blocks, but responded strongly to Stop-relevant Stop-trials. In all these areas the Stop-relevant Stop-trials yielded significantly stronger activations than the Stop-irrelevant Stop-trials (all p<0.05; see Fig. 4 for further significant differences). Comparisons among only the Stop-relevant Stop-trials indicate that the right IFG displayed a trend for stronger activity for successful than for unsuccessful Stop-trials (t(14) = 2; p = 0.07). Interestingly, the pre-SMA showed a significant effect in the opposite direction (t(14) = 2.4; p = 0.03). The right IFJ displayed a different general activity profile, with stronger activity for successful Stop-trials than for unsuccessful Stop-trials (t(14) = 2.9; p = 0.01) or for Stop-irrelevant Stop-trials (t(14) = 2.4; p = 0.03), but the average of all Stop-relevant Stop-trials did not differ significantly from Stop-irrelevant Stop-trials in this area (p>0.3).

## Discussion

The present fMRI study aimed at delineating the neural processes that are involved in the context of response inhibition during the Stop-signal task and to distinguish different neural underpinnings of the various cognitive processes engaged during such tasks. In an attempt to identify areas that respond to the rare and salient sensory stimulation of Stop-trials in an automatic fashion, we found that occipital and inferior parietal areas respond more strongly to Stop-trials than to Go-trials, even if the Stop-stimuli are completely task-irrelevant. Interestingly, this analysis also identified clusters in the right IFJ and the right pre-SMA that responded in a similar fashion, albeit only on a comparatively lenient uncorrected significance level. An additional ROI analysis that focused on the comparison of neural responses to Stop-trials from task blocks in which the Stop-stimuli were versus were not task-relevant identified three major activity profiles for different cortical areas. These profiles indicate a hierarchy in which pure sensory processing is mostly restricted to occipital areas, whereas some degree of automatic attentional capture by rare Stop-stimuli regardless of their task-relevance occurs in the inferior parietal lobules. In contrast, in the third profile, activity in a wide range of frontal areas and the right TPJ/STG was prominent only for Stop-trials that were task-relevant. These findings provide an important step towards establishing a framework in which sensory processes, bottom-up attentional processes, and top-down control functions can be attributed to specific portions of the wider cortical network that is typically associated with response inhibition during the Stop-signal task.

### Visual stimulation, attention, and response inhibition

At least three recent publications have highlighted the difficulty of distinguishing processes directly involved in response inhibition from those related to the attentive processing of the relevant stimuli, focusing on the role of right IFG ([22]–[24], see also [25]–[29]). The majority of these studies concluded that the right IFG, which has typically been considered a crucial node in response inhibition, may only be indirectly related to this function, and that this area may actually be more generally involved in the attentive processing of the Stop-stimuli. Using functional connectivity patterns derived from Granger causality analyses of fMRI data, Duann and colleagues observed that the right IFG influenced activity in the motor system only indirectly via the pre-SMA ([24], see also [28]). They concluded that the connectivity pattern of the right IFG suggested an attentional role based on its close functional relationship with temporal and parietal brain structures (see also [40]). Two subsequent studies have made similar arguments by investigating modified Stop-signal tasks that used high-level control stimuli that did not require response inhibition [22], [23].

In addition to an attentional account of the function of the IFG, the ubiquity of its activation across different tasks might also relate to recent accounts that assign more global control functions to this area (for recent reviews, see [41], [42]). Moreover, it has recently been suggested in the context of a modified Go-NoGo task that the requirements for response control irrespective of response inhibition can also strongly activate the right IFG [29]. Specifically, these authors report strong activations of the right IFG (exceeding the activation level of NoGo trials) for a control condition where subjects had to press an additional button in response to a third class of stimuli that were equally infrequent as NoGo trials. Note, however, that there is still controversy about whether the right IFG is really not directly related to response inhibition [21]. Either way, the example of the right IFG thus highlights the importance of further distinguishing related functions on a brain-wide level, both for understanding the basic underlying cognitive functions and because psychopathological derailment of other functions could potentially mimic deficits in motor control (e.g., [34]). For example, a number of studies have reported that fluctuations in attentional engagement can strongly influence the outcome of Stop-trials in this task ([11]–[15]; see also [43]), thereby mimicking fluctuations in response-inhibition processes.

Importantly, none of the studies mentioned above included a condition in which Stop-stimuli were entirely task-irrelevant, thus leaving open the question whether the right IFG and other areas would respond to Stop-stimuli even when they are entirely task-irrelevant. Such activation could arise by means of bottom-up attentional capture by the Stop-stimuli simply due to their rarity and physical salience. The present study identified activation by task-irrelevant Stop-stimuli bilaterally in occipital areas and in IPL, as well as in the right IFJ and pre-SMA (albeit on a more lenient, uncorrected significance threshold). Other frontal areas such as the IFG, however, did *not* respond in this condition, thus indicating that their task involvement depends on some level of task-relevance of the Stop-stimuli. This relevance, in turn, does not have to be based on the necessity to withhold a motor response, but at a minimum on the requirement that such a stimulus needs to be discriminated from the other stimuli in the sequence to determine its task-relevance.

Thus, the area that appeared to be most consistently activated by the mere rarity and salience of Stop-stimuli, irrespective of their task relevance, was the bilateral IPL, thus arguing against notions that have ascribed a direct involvement of this area in response inhibition (e.g., [44]). Activity in the IPL was highly similar in response to task-relevant and task-irrelevant Stop-stimuli, even when directly comparing activity estimates in the ROI analysis (i.e., avoiding conservative voxel-wise tests), thus also arguing against a graded involvement in the sense of a weak involvement in task-irrelevant Stop-trials that is enhanced for task-relevant ones. Based on this activity pattern, we would suggest that the IPL’s role would be described in terms of automatic attentional capture by rare salient events. Given this, it is slightly surprising that there is in fact no *behavioral* effect of attentional capture for task-irrelevant Stop-trials (i.e., no RT decrement as compared to the corresponding Go-trials). Nevertheless, the IPL appears to be mostly responsive to Stop-trials, thus arguing against a simple sensory role. However, it is not entirely atypical to find neural indications of attentional capture in the absence of a significant behavioral effect (e.g., [45]). Moreover, independent of its precise function, it appears that IPL is fulfilling a role during the Stop-signal task that is quite exclusive to Stop-trials, yet not directly related to response inhibition.

For areas that respond to Stop-trials only when they are task-relevant, however, response inhibition processes cannot be easily distinguished from those related to enhanced attentive processing of the Stop-stimuli, or those related to increased response control demands that are not inhibitory (e.g., [29]). This paradigmatical problem is difficult to overcome if it is the case that task-relevant Stop-stimuli require more attention or other top-down control mechanisms than control stimuli that require a different response. Consequently, areas that are more active during task-relevant Stop-trials could generally subserve several different functions. The present data suggest that for most of the frontal activations, as well as for those in the TPJ/STG, Stop-stimuli do not elicit a robust neural response if they are completely task-irrelevant. However, while this defines a “lower limit” of basic processes that do not elicit activity in typical response-inhibition areas, the present data cannot further distinguish between different high-level operations such as top-down attentional engagement, response demand complexity, and response inhibition.

### The role of right IFJ and MFG

While the right IFG and pre-SMA have commonly been discussed in the context of response inhibition, other frontal areas such as the right IFJ, MFG, and left anterior insula have also been frequently implicated in such tasks. Considerably less is known about the functional role of these other areas, however. For example, the right IFJ has been argued to play a role in detecting infrequent NoGo-trials in a Go-NoGo task, including because it has been shown to also respond to an additional type of Go-trials during a Go-NoGo task when they are presented as infrequently as the NoGo-trials [25]. This finding dovetails with our observation that the right IFJ also responds to Stop-trials that are entirely task-irrelevant (see also [46]). The present data furthermore revealed that the right IFJ was more active during successful than during unsuccessful Stop-trials (with the latter triggering similar levels of activity as the task-irrelevant Stop-trials), echoing the results from another recent report [47]. Interestingly, it has also been reported that the right IFJ is more active for Go-trials that *might* turn into a Stop-trial than for Go-trials that could not do so (“conditional” Stop-signal task, see [48]), suggesting that such activations may represent a process related to the preparation to inhibit a response [49], rather than being part of the inhibition-generating processes itself (see also [50]). More generally, the right IFJ has been implicated in maintaining, updating, and/or activating task sets [51], [52]. Our finding that the right IFJ activity is enhanced during successful versus unsuccessful Stop-trials suggests that it may play a role either specifically in preparing response inhibition, or in representing and enforcing the task rules more generally to influence the outcome of Stop-trials.

The current results also revealed a large cluster of activity in the right MFG during task-relevant Stop-trials, in line with results from earlier response-inhibition paradigms [9], [25], [53]–[55]. In general, the role of this area during response inhibition has not been well characterized, but it has been suggested to be involved in task-related, top-down control processes [56], potentially related to working memory demands [27]. Given that the present MFG activation did not differentiate between successful and unsuccessful Stop-trials, we cannot attribute a more specific role to it, beyond the fact that it only responds to Stop-trials when they are task-relevant.

### The cortical control of response inhibition

Given recent reports of right IFG activation by control stimuli that do not require response inhibition (e.g., [22], [23]), it is possible that the role of this brain area in response inhibition may be relatively indirect, which would be counter to most earlier notions that identified it as being critical for this function. Accordingly, it would not be clear which cortical area, if not the right IFG, actually initiates the cancellation of a motor response. Although one possible candidate is the right pre-SMA (e.g., [23], [24]), it is noteworthy that in our study, as well as in another recent report [22], the pre-SMA was *more* active during unsuccessful than during successful Stop-trials. This raises the question whether inhibition-related activity is really stronger in successful than in unsuccessful Stop-trials [35], as is usually assumed. Alternatively, it is conceivable that other control-modulating factors, such as fluctuations in the perceptual/attentional processing of the task-relevant stimuli, are critical in determining the outcome of the process, while activity fluctuations related to actual response inhibition may not be the critical determining factor as to whether a Stop-trial is responded to successfully or not. Another possibility would be that the fMRI signal, due to its low temporal resolution, additionally includes processes that occur in the pre-SMA before or after the response has been (or has not been) generated, which could also differ between the successful and unsuccessful Stop-trials without reflecting response inhibition processes. The fact that the pre-SMA might perform additional operations during this task that are not related to response inhibition is further supported by our finding that there appears to be a weak pre-SMA response even for task-irrelevant Stop-trials. Although it is important to note that the respective cluster was only weakly activated and did not survive multiple-comparison correction, the fact that the peak coordinate was identical to the one that resulted from the analysis that also included task-relevant Stop-trials would appear to give some more credibility to this activation. Based on the observation that the pre-SMA generally tracks response speed in this context, even for Go-trials (e.g., [57]), it is possible that this activity is related to the small amount of slowing that occurs during task-irrelevant Stop-trials (as compared to the respective Go-trials). Nonetheless, other functions are conceivable, including for example a role in attentional shifting [30]. Regardless, the current findings underscore that further research is needed to more clearly disentangle the functional components of processes engaged during the inhibition of a motor output in response to the detection of a unique stimulus type in a stream of other stimuli.
